# Multiple health and environmental impacts of foods

**DOI:** 10.1073/pnas.1906908116

**Published:** 2019-10-28

**Authors:** Michael A Clark, Marco Springmann, Jason Hill, David Tilman

**Affiliations:** ^a^Oxford Martin Programme on the Future of Food, University of Oxford, OX3 7LF Oxford, United Kingdom;; ^b^Nuffield Department of Population Health, University of Oxford, OX3 7LF Oxford, United Kingdom;; ^c^Natural Resources Science and Management, University of Minnesota, St. Paul, MN 55108;; ^d^Department of Bioproducts and Biosystems Engineering, University of Minnesota, St. Paul, MN 55108;; ^e^Department of Ecology, Evolution, and Behavior, University of Minnesota, St. Paul, MN 55108;; ^f^Bren School of Environmental Science and Management, University of California, Santa Barbara, CA 93106

**Keywords:** food, health, environment, diet, climate change

## Abstract

Food choices are shifting globally in ways that are negatively affecting both human health and the environment. Here we consider how consuming an additional serving per day of each of 15 foods is associated with 5 health outcomes in adults and 5 aspects of agriculturally driven environmental degradation. We find that while there is substantial variation in the health outcomes of different foods, foods associated with a larger reduction in disease risk for one health outcome are often associated with larger reductions in disease risk for other health outcomes. Likewise, foods with lower impacts on one metric of environmental harm tend to have lower impacts on others. Additionally, of the foods associated with improved health (whole grain cereals, fruits, vegetables, legumes, nuts, olive oil, and fish), all except fish have among the lowest environmental impacts, and fish has markedly lower impacts than red meats and processed meats. Foods associated with the largest negative environmental impacts—unprocessed and processed red meat—are consistently associated with the largest increases in disease risk. Thus, dietary transitions toward greater consumption of healthier foods would generally improve environmental sustainability, although processed foods high in sugars harm health but can have relatively low environmental impacts. These findings could help consumers, policy makers, and food companies to better understand the multiple health and environmental implications of food choices.

## Significance

Dietary choices are a leading global cause of mortality and environmental degradation and threaten the attainability of the UN’s Sustainable Development Goals and the Paris Climate Agreement. To inform decision making and to better identify the multifaceted health and environmental impacts of dietary choices, we describe how consuming 15 different food groups is associated with 5 health outcomes and 5 aspects of environmental degradation. We find that foods associated with improved adult health also often have low environmental impacts, indicating that the same dietary transitions that would lower incidences of noncommunicable diseases would also help meet environmental sustainability targets.

Dietary choices—the types and amounts of foods that individuals consume—are a major determinant of human health and environmental sustainability. Nine of the top 15 risk factors for global morbidity result from poor dietary quality, while diseases associated with poor dietary quality, including coronary heart disease (CHD), type II diabetes, stroke, and colorectal cancers, account for nearly 40% of global mortality (1, 2). Furthermore, agricultural food production emits ∼30% of global greenhouse gasses (GHGs) (3, 4); occupies ∼40% of Earth’s land (5); causes nutrient pollution that profoundly alters ecosystems and water quality (6); and accounts for ∼70% of Earth’s freshwater withdrawals from rivers, reservoirs, and ground water (7), among other negative environmental effects (8, 9).

Here we examine the potentially complex and multifaceted food-dependent linkages between and among 5 different diet-dependent health outcomes in adults—type II diabetes, stroke, coronary heart disease, colorectal cancer, and mortality—and 5 different environmental impacts of producing the foods. Such information could help consumers, food corporations, and policy makers make better decisions about food choices, food products, and food policies, potentially increasing the likelihood of meeting international sustainability targets such as the United Nations’ Sustainable Development Goals or the Paris Climate Agreement (10, 11). Previous analyses have examined the overall health and environmental impacts of dietary patterns (e.g., refs. 12 and 13), but have not decomposed these multifaceted impacts to individual foods at quantities consumed on a daily basis. Moreover, analyses looking at individual foods commonly examine the health (e.g., ref. 14) or environmental impacts (e.g., ref. 15) in isolation of the other.

In particular, we explore the multiple human health and environmental impacts of 15 different food groups: chicken, dairy, eggs, fish, fruits, legumes, nuts, olive oil (which we include as an indicator for vegetable oils high in unsaturated fatty acids because of data availability; see the discussion in *SI Appendix*), potatoes, processed red meat, refined grain cereals, sugar-sweetened beverages (SSBs), unprocessed red meat, vegetables, and whole grain cereals. Our analysis includes the 5 health outcomes mentioned above and 5 environmental outcomes—GHG emissions, land use, scarcity-weighted water use (water use multiplied by a constant that scales regionally based on water availability after demand from humans and aquatic ecosystems has been met) (16), and 2 forms of nutrient pollution—acidification and eutrophication. We first consider the health and environmental impacts of these foods separately, and then explore them jointly.

We selected these foods and these health and environmental outcomes because plausible causal metabolic mechanisms between food consumption and health outcomes exist for these foods and because the health and environmental impacts of these foods have been well documented through metaanalyses. The health outcomes reported here are the relative risks (RRs) of disease resulting from consuming an additional serving of a food per day relative to the average intake of that food observed in a cohort study. If RR > 1, consumption of an additional serving is associated with increased disease risk compared to the average risk of that disease, and if RR < 1, this consumption is associated with decreased disease risk. The food-dependent health data are from 19 dose–response metaanalyses (see *SI Appendix*, Table S1, for complete list) (17–35), which follow adult populations through time to estimate how food consumption is associated with disease risk while statistically controlling for confounding factors such as age, body mass index, sex, and history of smoking. Infants and children may have different nutritional needs. The 5 environmental outcomes reported here are the impacts of producing a serving of each food group as estimated by metaanalyses of life cycle assessments (LCAs) that account for the environmental impacts of plant and animal production, including the production, manufacture, and use of agricultural inputs, seed, equipment, and cropland (15), but not transport, processing, retail, and food preparation. Because most food groups contain multiple foods, the environmental impact per serving of each food group is weighted by the global average consumption of the foods within each food group (5).

## Results

### Health Outcomes of Food Groups.

**Fig. 1.**
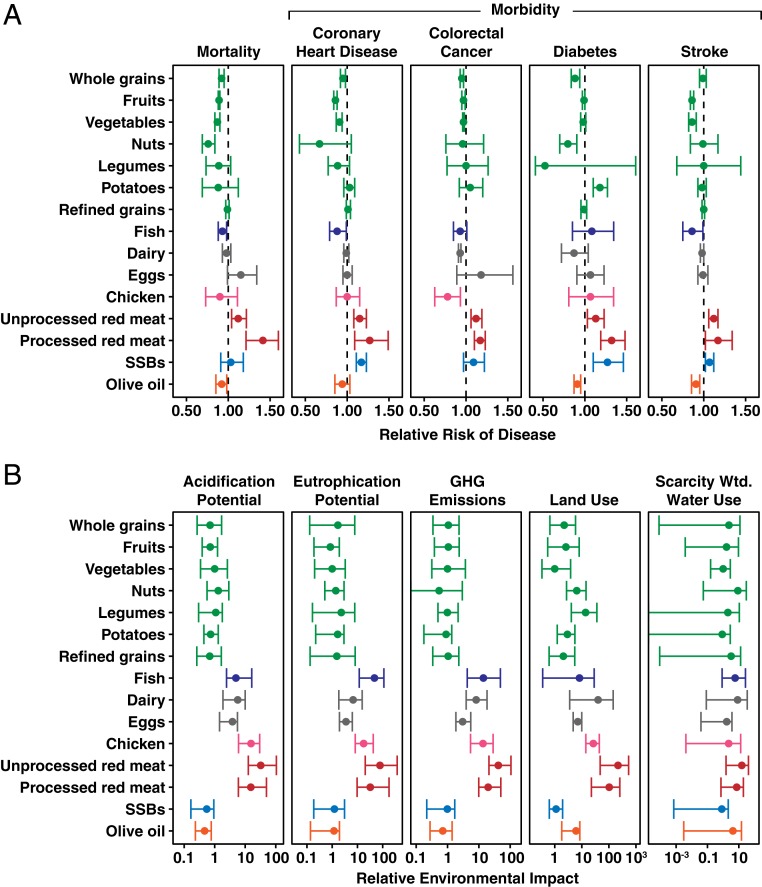
Summary of health and environmental data. (*A*) Health data are reported as the RR of disease per serving of food consumed, where an RR <1 indicates that food consumption is associated with decreased disease risk and an RR >1 indicates that food consumption is associated with increased disease risk. Error bars for the health data indicate the 5th and 95th percentile confidence intervals. (*B*) Environmental data are shown as the relative environmental impact per serving of food produced, where a value of 1 indicates that producing a serving of food has the same environmental impact as producing a serving of vegetables. Environmental impacts are plotted on a log10 scale, and error bars for the environmental data indicate the 5th and 95th percentile impacts per serving of food produced. Water use is reported as scarcity-weighted (Wtd) water use, which accounts for regional variation in water availability. Data used to create the plots are available in Dataset S1. The association between total mortality and olive oil was estimated by weighting disease-specific contributions (e.g., CHD, stroke, and diabetes) to mortality by disease-specific relative risk (2).

We found few tradeoffs among the health impacts of different foods. In particular, no food associated with a significant (at *P* < 0.05) reduction in disease risk for one health outcome was associated with a significant increase in disease risk for any other health outcome (Fig. 1). Indeed, Spearman rank-order correlations showed that a food group that benefitted (or harmed) one health metric tended to have similar affects on the other health metrics. In particular, we determined the rank-order correlation for each of the 10 pairwise comparisons of the 5 health impacts. Each correlation used data for the 15 food groups. We found that 8 of these 10 Spearman correlations were significant and positive (*P* < 0.05; *SI Appendix*, Table S2), while none were significant and negative.

As to individual food groups, nuts, minimally processed whole grains, fruits, vegetables, legumes, olive oil, and fish are associated with significantly (*P* < 0.05) reduced mortality and/or reduced risk for one or more diseases (Fig. 1*A*). Consuming an additional serving per day of these 7 foods is associated with a significant reduction in risk for 20 of the 34 health endpoints for these foods (7 foods by 5 health outcomes; dose–response data for the association between olive oil and colorectal cancer was not available) and no significant change in disease risk for 14 of 34 health outcomes. We note, however, that because the health benefit of increasing consumption of these 7 foods is often nonlinear, the health benefit of consuming a second additional serving per day is often smaller than the health benefit of consuming the first additional serving per day. While data from dose–response cohort metaanalysis were available for olive oil but not for other vegetable oils that are similarly high in unsaturated fatty acids and low in saturated fats, other types of health analyses suggest that other such vegetable oils might have health benefits similar to those of olive oil (36).

Daily consumption of an additional serving of dairy, egg, and chicken is not significantly associated with disease incidence for 12 of the 14 health endpoints (Fig. 1*A*; 3 foods by 5 health outcomes; dose–response data for chicken and stroke were not available). However, the inability to fully control for potential dietary confounders (e.g., reduced consumption of red meat when chicken consumption increases) likely influences the observed associations between consumption of chicken and disease risk in particular, and between food consumption and health outcomes more generally (33). Similarly, consuming an additional 30 g of refined grain cereals was also not associated with a significant change in disease incidence, although consuming larger amounts of refined grain cereals has been associated with increased risk of diabetes. Substituting whole grain cereals for refined grain cereals has been associated with reductions in disease incidence (37, 38).

Consumption of sugar-sweetened beverages, unprocessed red meat, and processed red meat are consistently associated with increased disease risk (Fig. 1*A*). Sugar-sweetened beverage consumption is associated with a significant increase in CHD, type II diabetes, and stroke, but not total mortality or colorectal cancer. Consumption of unprocessed and processed red meat is associated with significant increases in disease risk for all 5 health outcomes examined here. Of all of the foods examined, a daily serving of processed red meat is associated with the largest mean increase in risk of mortality and incidences of CHD, type II diabetes, and stroke.

The health outcomes reported here were estimated by tracking the dietary patterns and health outcomes of tens of millions of individuals. While individuals of a wide variety of ethnicities, ages, and economic statuses who consumed a diverse array of dietary patterns were included in the primary analyses, the majority of individuals included in these studies likely ate Westernized diets since they lived in higher-income countries such as those in Europe, the United States, or Canada, and a smaller number in Asian countries and other regions (*SI Appendix*, Table S4). As such, the health outcomes reported here are most relevant and applicable to individuals whose diets and lifestyles are similar to those typically found in higher-income regions (e.g., high in calories, sugar, highly refined foods and animal source foods, and low in whole grains). It is possible that eating just a single daily serving of a food may have quantitatively different health implications than we report for eating an additional serving beyond the mean number eaten daily in Westernized diets. In addition, the health outcomes reported here control for body mass index. As such, the potential health implications of consuming an additional serving of one food without reducing consumption of another food (i.e., thereby leading to increased calorie intake and possibly weight gain) are not included in the health estimates reported here despite the known health implications of excess caloric consumption (25).

### Environmental Outcomes of Food Groups.

The mean GHG emissions, land use, acidification, and eutrophication per serving of food produced for the 15 food groups differed by 2 orders of magnitude (Fig. 1*B*). As illustrated by the 95% confidence intervals around each mean shown in Fig. 1*B*, for most food groups and most environmental impacts, there is about a 10-fold difference between the lower and upper confidence interval values. To the extent that this variation reflects the effects of different methods of crop production, marked improvements in environmental impacts may be possible for most foods. While mean scarcity-weighted water use per serving of food produced did not significantly vary across these 15 foods, unprocessed red meat had twice the water impact of dairy, nuts, processed red meat (which has a smaller serving size than unprocessed red meat), and olive oil, which in turn had more than twice the impacts of the remaining foods. This general pattern, and the large variation around the mean scarcity-weighted water use, merits further exploration.

To better examine similarities across different environmental indicators, we report all environmental impacts relative to the impact of producing a serving of vegetables, that is, as the ratio of the impact of producing a serving of a given food divided by the impact of producing a serving of vegetables. When looking across the different environmental indicators, we found that foods that have a low mean relative environmental impact per serving for 1 environmental indicator often also have low mean relative environmental impacts for the other 4 environmental indicators. Indeed, Spearman rank-order correlations for 9 of the 10 pairwise comparisons between the 5 types of environmental impacts are positive and significant (*P* < 0.05; *SI Appendix*, Table S2); only the association between GHG emissions and scarcity-weighted water use is nonsignificant (*P* = 0.145). Minimally processed plant source foods, olive oil, and sugar-sweetened beverages consistently have among the lowest environmental impacts for all indicators, often having a relative environmental impact of less than 5 for all 5 environmental indicators. Dairy, eggs, fish, and chicken have relative environmental impacts that range from 3 to 40 for GHGs, acidification, eutrophication, and land use. Producing a serving of unprocessed red meat has the highest impact for all 5 environmental indicators, with a relative environmental impact ranging from 16 to 230. Producing a serving of processed red meat has the second highest mean impact on acidification, GHG emissions, and land use and the third highest mean impact for eutrophication. In our analysis, we weighted food production impacts based on global production location and methodology to arrive at an average global estimate of the environmental impact per unit of food produced. While our environmental data primarily come from LCAs, other methodologies estimating the environmental impacts of producing different foods show that while the environmental impacts of food production per unit produced varies across regions, the relative rankings of the environmental impacts of producing different foods is often similar (39, 40).

### Combining Health and Environmental Outcomes.

**Fig. 2.**
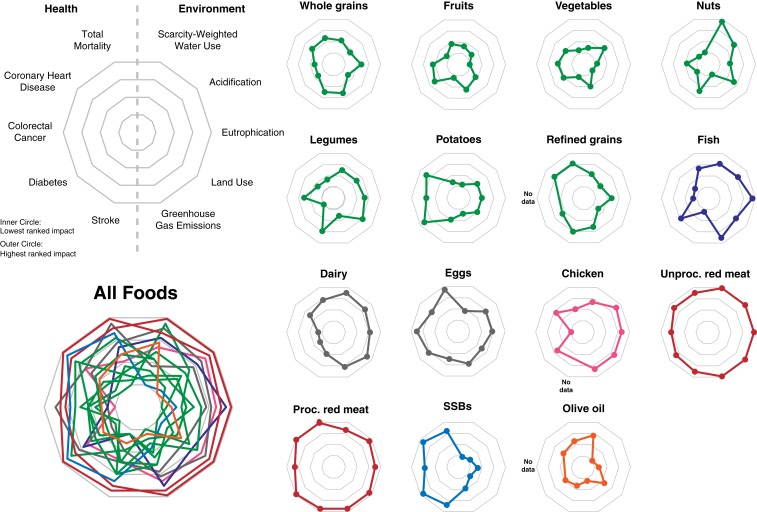
Radar plots of rank-ordered health and environmental impacts per serving of food consumed per day. Data are plotted on a rank order axis such that the food group with the lowest mean impact for a given health or environmental indicator (lowest is best health or environmental outcome) has a value of 1 (innermost circle), and the food group with the highest mean impact for a given indicator has a value of 15 (outermost circle). The *Left* side of each radar plots shows health outcomes; the *Right* side shows environmental impacts. A food group with low mean impacts for the 10 outcomes would have a small circular radar plot, and one with high impact for the 10 outcomes would have a large circular radar plot. The “all foods” radar plot combines data for the 15 food groups into a single plot. Data used to create the plot are available in Dataset S1. SSBs are sugar-sweetened beverages. The association between total mortality and olive oil was estimated by weighting disease-specific contributions (e.g., CHD, stroke, and diabetes) to mortality by disease-specific relative risk (2).

Combining all data into a “radar plot” for each food facilitates comparison across the multiple health and environmental impacts of each food (Fig. 2 and *SI Appendix*, Fig. S1). Plotting the 5 health and 5 environmental impacts of each food on quantitatively ranked axes, where points closer to the origin are healthier or have lower relative environmental impact, shows that foods with among the lowest environmental impacts often have the largest health benefits (lowest relative risks of disease or mortality), and that the foods with the largest environmental impacts—unprocessed and processed red meat—often have the largest negative impacts on human health. These patterns are particularly clear when foods are ranked by each of the health or environmental impacts (Fig. 2), but are also apparent when the absolute impacts are plotted (*SI Appendix*, Fig. S1). Producing a serving of unprocessed and processed red meats has environmental impacts 10 to 100 times larger than those of plant source foods for GHG emissions, land use, acidification, and eutrophication (Fig. 1*B*).

The variation around the mean health and environmental impacts (Fig. 1 *A* and *B*) can result from differences among foods within each food group, food preparation, or production methodology. For instance, consumption of leafy green vegetables has been associated with a significant reduction in type II diabetes risk, whereas some other vegetables have not (14). Similarly, per unit of food produced, rice production emits more GHGs than other cereals because methane is produced when rice paddies are flooded. For red meats, ruminant meat (beef, sheep, and goat) has higher environmental impacts than pork because ruminant meat production uses more agricultural inputs than pork per unit of meat produced and because ruminants emit methane when digesting food (15). For health (Fig. 1*A*) but not for environmental (Fig. 1*B*) impacts, variation around the mean also results from differences in food preparation method. For instance, frying fish can negate its potential health benefits (17). For environmental but not for health impacts, variation can also result from differences in production location or methodology. For instance, the GHG emissions of fish production are highly variable, in part because of the variety of fish production methods. Bottom trawling fisheries and recirculating aquaculture systems emit more GHG per amount of fish produced than do other fish production system because of greater energy use (41). Further description and explanation of the variation around the mean impact for each food group is in *SI Appendix*.

### Associations between Health and Environmental Outcomes.

Finally, to look for broad and general associations between the health and environmental impacts of food types, we compare the diet-related relative risk of mortality of each food group to the group’s averaged relative environmental impact (AREI, the average of a food’s relative environmental impact per serving across all 5 environmental indicators).

**Fig. 3.**
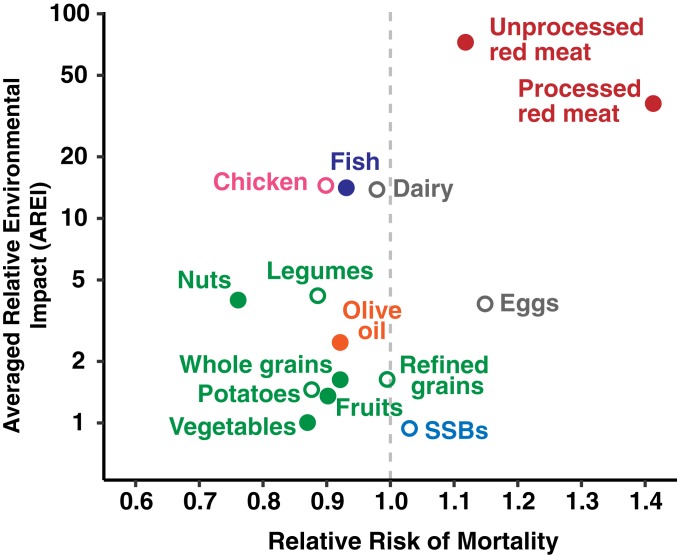
Association between a food group’s impact on mortality and its AREI. The *y* axis is plotted on a log scale and is the AREI of producing a serving of each food group across 5 environmental outcomes relative to the impact of producing a serving of vegetables (not including starchy roots and tubers). The *x* axis is the relative risk of mortality, where a relative risk >1 indicates that consuming an additional daily serving of a food group is associated with increased mortality risk, and a relative risk <1 indicates that this consumption is associated with lowered mortality risk. Labels and points are colored with green = minimally processed plant-based foods; dark blue = fish; gray = dairy and eggs; pink = chicken; red = unprocessed red meat (beef, lamb, goat, and pork) and processed red meat; light blue = sugar-sweetened beverages; and orange = olive oil. Food groups associated with a significant change in risk of mortality (at *P* < 0.05) are denoted by solid circles. Food groups not associated with a significant change in mortality risk are denoted by open circles. Serving sizes for the food groups are: whole grains (30 g dry weight); refined grains (30 g dry weight); fruits (100 g); vegetables (100 g); nuts (28 g); legumes (50 g dry weight); potatoes (150 g); fish (100 g); dairy (200 g); eggs (50 g); chicken (100 g); unprocessed red meat (100 g); processed red meat (50 g); SSBs (225 g); and olive oil (10 g). Data used to create the plot are available in Dataset S1. The association between total mortality and olive oil was estimated by weighting disease-specific contributions (e.g., CHD, stroke, and diabetes) to mortality by disease-specific relative risk (2).

Foods associated with significant reductions in mortality consistently have a low averaged relative environmental impact (Fig. 3). Whole grain cereals, fruits, vegetables, nuts, and olive oil have an AREI of 4 or less per serving. Fish, the other food group that is associated with a significant reduction in mortality, has an AREI of 14 per serving. Foods associated with a significant increase in mortality have variable environmental impacts (Fig. 3). Unprocessed red meats (AREI = 73) and processed red meats (AREI = 37) have the highest AREIs while sugar-sweetened beverages (AREI = 0.95) have the lowest AREIs of all foods in this analysis. Qualitatively similar relationships occur between AREI and the food-dependent relative risks of diabetes, CHD, stroke, and colorectal cancer (*SI Appendix*, Fig. S2).

## Conclusions

The same dietary changes that could help reduce the risk of diet-related noncommunicable diseases could also help meet international sustainability goals. Focusing diets on foods consistently associated with decreased disease risk would likely also reduce diet-related environmental impacts. Foods with intermediate environmental impacts or that are not significantly associated with health outcomes, such as refined grain cereals, dairy, eggs, and chicken, could also contribute to meeting international health-focused or environmental-focused sustainability targets if they are used to replace foods that are less healthy or have higher environmental impacts such as unprocessed red meat and processed red meat (42).

Other foods, such as trans fats, ultraprocessed foods, and added sugar, were not included in this analysis because no dose–response metaanalyses had examined the association between consumption of these foods and health outcomes. However, health analyses using different methodologies have linked consumption of trans fats and ultraprocessed foods with increased disease risk (35, 43). Furthermore, added sugar consumption has been associated with an increase in risk of cardiovascular disease (44), but has not been associated with increased risk of total mortality in individual cohort studies (45), although this may be because cohort studies often control for body weight, and the impact of added sugar consumption on risk of total mortality is at least partially caused by weight gain. Added sugars tend to have lower environmental impacts, as do ultraprocessed foods if they contain no or small amounts of animal source foods (15).

Food consumption and production are directly linked with other aspects of human health and environmental degradation beyond those included in this analysis. The data we used do not address the impacts of nutrition on child development. For instance, vitamin A deficiency resulting from poor dietary quality is a major source of poor eyesight, blindness, and childhood mortality in developing regions, while reduced air quality resulting from food production is responsible for ∼20% of deaths from air pollution (9, 46). Similarly, food production is the largest stress to biodiversity through habitat destruction and nutrient pollution, with food production threatening >70% of birds and mammals that are listed as threatened with extinction by the International Union for Conservation of Nature (IUCN) (47).

Global diets have been shifting toward greater consumption of foods associated with increased disease risk or higher environmental impacts and are projected to lead to rapid increases in diet-related diseases and environmental degradation (12, 13, 48–50). Reversing this trend in the regions in which it has occurred and instead increasing consumption of whole grain cereals, fruits, vegetables, nuts, legumes, fish, and olive oil and other vegetable oils high in unsaturated fats—foods that are consistently associated with decreased disease risk and low environmental impacts—would have multiple health and environmental benefits globally. Public and private solutions could help shift food consumption toward healthier and more environmentally sustainable outcomes.

## Methods

We first analyzed the impact on adult health of consuming an additional serving of food per day (1 serving more than the cohort average) for 15 food groups. In particular we synthesized results from 19 recent dose–response metaanalyses to determine how 5 health outcomes—incidences of colorectal cancer, CHD, type II diabetes, and stroke, as well as risk of total mortality—were impacted by consuming an additional serving of each type of food per day (see *SI Appendix*, Table S1, for the dose–response metaanalyses included in this analysis and *SI Appendix*, Table S3, for the serving sizes reported by the dose–response metaanalyses). We limited our analyses to these 15 food groups because dose–response metaanalyses for these foods were available. The existence of dose–response relationships from multiple cohorts, together with plausible pathways that explain the change in disease risk, suggest that the risk relationships are reflective of biological processes and are broadly applicable. Because there were no dose–response metaanalyses examining the association between olive oil consumption and risk of total mortality, we estimated this association by weighting disease-specific contributions (e.g., CHD, stroke, and diabetes) to mortality by disease-specific relative risk (2).

We then determined, for each of the 15 food groups, how agricultural production of a serving of each food impacted 5 types of environmental degradation—GHG emissions, land use, scarcity-weighted water use, and acidification and eutrophication (2 forms of nutrient pollution)—using data from recent life cycle metaanalyses (15, 41). While data from life cycle metaanalyses are primarily from high-income and high-input nations, other methodologies of estimating the environmental impacts of food production have shown that while the environmental impacts of food production per unit of food produced varies across regions, the relative rankings of the environmental impacts of different foods is similar across regions (39, 40). Using metaanalyses of LCAs can be considered more reliable and reflective of the general magnitudes of environmental impacts of different foods than individual LCAs because of potential variation between individual LCAs.

To better allow broad comparisons between the overarching health and environmental impact of different foods, we also calculated the averaged environmental impact of each food by first calculating the impact of producing a food for each indicator relative to the impact of producing vegetables. The averaged relative environmental impact was then calculated as the average of the relative impacts for the 5 environmental outcomes examined here. As such, a food group with an averaged relative environmental impact of 5 indicates that producing a serving of that food group results, on average, in 5 times the environmental impacts across the 5 environmental outcomes examined here than does producing a serving of vegetables.

The serving sizes used in this analysis are 225 g for sugar-sweetened beverages; 200 g for dairy; 150 g for potatoes; 100 g for chicken, red meat, fish, fruits, and vegetables; 50 g for processed red meat, eggs, and legumes; 30 g for refined grains and whole grain cereals; 28 g for nuts; and 10 g for olive oil. In cases where dose–response metaanalyses reported health outcomes at different serving sizes, we calculated the reported RR of disease risk for the aforementioned serving sizes by accounting for linearities and nonlinearities in the association between food consumption and disease risk.

### Statistics.

Statistics in the scope of this study are reported in 2 ways. First, associations between food consumption and health outcomes are reported as “significant” if the association is reported as having a *P* value <0.05 in the relevant dose–response metaanalysis. Second, significant associations between pairwise Spearman ranked correlations for the health and environmental outcomes were tested using the function “rcorr” from the package “Hmisc” in R. Data used for the Spearman ranked correlations and associated *P* values are in *SI Appendix*, Table S2.

### Data Availability.

All data used in this study are available in Dataset S1 and *SI Appendix*, Tables S1–S4.

## Supplementary Material

Supplementary File

Supplementary File
