# Supplementary material for: Multiple health and environmental impacts of foods
Source: Proc Natl Acad Sci U S A. 2019 Oct 28;116(46):23357–62. doi: 10.1073/pnas.1906908116 (PMC6859310; doi:10.1073/pnas.1906908116)
Supplement: Supplementary File [file pnas.1906908116.sapp.pdf]

Supplementary Information for

Multiple health and environmental impacts of foods

Michael A Clark[1,2], Marco Springmann[2], Jason Hill[3], David Tilman[4,5]

This PDF file includes:

Supplementary text
Fig. S1
Fig. S2
Tables S1 to S3
References for SI reference citations

Other supplementary materials for this manuscript include the following:

Datasets S1

Supplementary Information Text

Materials and Methods

Health

Description of dose-response meta-analyses, and data sources:

Prospective cohort studies follow populations through time as a way to examine the health outcomes of changes in risk factors, such as consumption of different foods or dietary patterns. Prospective cohort studies analyze health outcomes in one of three ways: 1) dose-response analyses; 2) quintile analyses; or 3) substitution analyses. Dose-response analyses report the health impact of consuming a serving of food per day, for example, the health impact of consuming an additional serving of red meat per day. Studies comparing quintiles of consumption often report the health impact of extreme quintiles, for example, the health outcome of the subgroup that consumes the least red meat against the health outcome of the subgroup that consumes the most red meat. Studies examining food substitution report the health outcome of substituting one food for another, for example the health outcome of substituting one serving of red meat per day with an equivalent amount of chicken per day.

Meta-analyses use data from numerous individual studies to derive a more general relationship and to reduce the risk of bias that might be present in any individual study. In our health analysis, we used meta-analyses that used data from individual cohorts to derive dose-response relationships that are believed to indicate relationships between food consumption and disease risk, and are also supported by the existence of plausible pathways that explain risk mediation.

We used dose-response meta-analyses analyses in this analysis for several reasons. First, they allow for more direct comparison of the health and environmental outcome of different foods in quantities that might be consumed at a single meal. For instance, the serving sizes reported in dose-response meta-analyses vary

www.pnas.org/cgi/doi/10.1073/pnas.1906908116

from 20 – 200g per day and are often similar in size to what is consumed at a meal (see Table S3 in the SI Appendix for serving sizes as reported in the dose-response meta-analyses). In addition, there are dose-response meta-analyses for most commonly consumed food groups. In total, we collected data from 19 dose-response meta-analyses that examined the health impact of consuming an additional serving of food per day for 15 food groups (17–35) (see Table S1 in the SI Appendix for publication list).

Dose-response meta-analyses control for confounding variables when reporting the health outcomes of food consumption. For instance, age, sex, history of smoking, race, and economic status are commonly controlled for in meta-analyses because they are known to influence health outcomes. Many dose-response meta-analyses report the health outcomes of consuming an additional serving of food per day when controlling for different amounts of confounding variables. When the outcomes of analyses with different amounts of confounding variables were reported, we used the health outcome from the analysis that controlled for the largest number of confounding variables to minimize the potential impact that uncontrolled confounding variables may have on the health outcomes of food consumption. In addition, we chose the dose-response meta-analysis that was most recently published when there were multiple dose-response meta-analyses examining the same food because these analyses often contained more studies and more individuals, and are thus more likely to represent the real health impact of consuming an additional serving of food per day. Dose-response meta-analyses that were funded in part by industry were not included in this analysis.

Many of the dose-response meta-analyses included in this analysis examine populations that are primarily Caucasian. However, the health impact of food consumption can differ depending on food preparation method (17), between individuals without and with pre-existing diseases (51), or between individuals that have different baseline dietary habits (e.g., ref (20)). For example, type II diabetes incidence is higher in men than women in Chinese, South Asian, and white populations (52), whereas African Americans have higher cancer rates for many cancers than Hispanics, Asian Americans, and Caucasians (53). However, while the results of the dose-response analyses included here are primarily based on Caucasian populations, the causal mechanisms of the health impacts of food consumption are assumed to hold irrespective of ethnicity. As such, it is unlikely that using analyses that examined non-Caucasian populations would have a large impact on the health outcomes reported here.

The underlying health data shows that consuming an additional serving per day of many of the foods examined here is associated with reduced disease risk. However, the marginal health benefit of increasing consumption of whole grain cereals, fruits, vegetables, nuts, and fish decreases as more of these foods are consumed (20, 22, 54, 55). Further, because excess caloric consumption and resultant weight gain lead to negative health outcomes (56), it is possible that eating more of a healthy food without decreasing consumption of other foods may not be beneficial to health. In addition, the dose-response health meta

analyses control for BMI, which means that the RR estimates used here do not account for the potential health impact of weight gain.

Estimating risk of mortality for olive oil

We estimated the association between olive oil consumption and risk of total mortality because there is not a dose-response meta-analysis examining this association. To estimate the mean RR, we weighted the RR risk for disease-specific endpoints based on their relative contributions to global mortality as estimated by the Global Burden of Disease (2). For example, because the mean RRs for olive oil consumption and risk of CHD, diabetes, and stroke are 0.94, 0.91, and 0.90, respectively, and the relative contribution of CHD, diabetes, and stroke to total mortality are 0.477, 0.095, and 0.428, respectively, the estimated mean RR for olive oil consumption and total mortality is thus 0.94 * .477 + 0.91 * 0.095 + 0.90 * 0.428, or 0.92. The estimated RR for the lower and upper confidence intervals were estimated in the same way, and are estimated to be 0.86 and 0.99, respectively.

Environment

Description of life cycle analyses and data sources

Life cycle assessment (LCAs) is a standardized method to estimate the environmental impacts per unit of food production. The meta-analysis of LCAs from which estimates of greenhouse gas (GHG) emissions, land use, nutrient runoff (specifically eutrophication) per gram of food were obtained estimated the environmental impacts from cradle to consumption (15). This system boundary accounts for all impacts that occur from pre-farm and on-farm activities such as fertilizer production and application, infrastructure construction, and on-farm fossil fuel, as well as post-farm activities such as transportation, processing, refrigeration, and cooking. GHG emissions from land-use change are also included in these estimates. Where possible, the estimates of environmental impacts per unit of food production were weighted nationally and internationally to be representative of current global average production location and methodology.

The impacts of producing a given food often differs in environmental impact across geographical regions and production methodology (39, 40). Using non-weighted estimates of the environmental impacts of producing a serving of food (e.g., estimates from certain locations or from certain types of production systems, such as organic) would change the estimates of the absolute impacts and relative impacts per serving of food produced. However, using non-weighted estimates would not change the broad rankings of the environmental impacts of producing a serving of different foods. That is, plant source foods would often have the lowest environmental impacts; dairy, chicken, and eggs would have intermediate environmental impacts; and unprocessed and processed red meat would have the highest environmental impacts (39, 40). Using results from existing meta-analyses that are weighted based on current global production methodologies rather than from individual studies is in line with the broad scope of our analysis

and reduces the risk of any potential data biases resulting from using data that is indicative of a single production location or methodology.

Scarcity-weighted water use is a metric that accounts for regional variation in water availability as well as the water used for food production (16). It is calculated by weighting the water used for food production by the amount of water available after accounting for water used in natural and agricultural processes. The southwest United States, Australia, the Middle East, Central Asia, Northern Africa, Southern Africa (South Africa, Namibia, Botswana, Mozambique, and Zimbabwe), and Chile have high scarcity water weightings. In contrast, Southeast Asia, New Zealand, Scandinavia, the eastern United States, Central America, northern South America (Colombia, eastern Ecuador, eastern Peru, Venezuela, Suriname, Guyana, French Guiana), and the Amazon Basin have low scarcity water weightings.

Agricultural production method

Agricultural production method can also influence a food's environmental impact. Producing organic foods, for example, often requires more land and causes more nutrient pollution per unit of food produced than non-organic systems (41, 57), whereas grass-fed beef tends to result in more GHG emissions and nutrient runoff than grain-fed beef (41). We controlled for agricultural production methodology by weighting the impacts per serving of food produced based on current global production from e.g. organic and conventional systems.

Estimating the environmental impacts of fish production

The environmental impact of fish production is highly dependent on production methodology. Trawling fisheries emit ~3 × more GHGs than other types of fisheries while recirculating aquaculture emits ~3 × more GHGs than non-recirculating aquaculture (41). Further, while production of wild-caught fish requires no land, uses no irrigation water, and results in very small amounts of eutrophication, production of fish in aquaculture systems requires land, often uses irrigation water, and results in larger amounts of eutrophication.

Because of the differences in the environmental impact of fish production, we estimated the environmental impact per serving of fish by first assuming that half of fish are produced in wild-caught fisheries and half of fish are produced in aquaculture, which is approximately equivalent to the current breakdown of global fish production (58). To estimate the impact of wild-caught fish, we further assumed that 20% of wild-caught fish are produced via bottom trawling or dredging and 80% are produced using other capture methodologies, which is in-line with estimates reported in Watson et al (2006).

Relative environmental impact

Because the absolute magnitude of the environmental impact of food production varies across environmental indicators, we reported the environmental impact in this analysis as the relative environmental impact, or the environmental impact relative to a serving of vegetables. As such, a relative environmental impact of 1 indicates that producing a serving of food has the same environmental impact as vegetables, a relative environmental of 0.5 indicates that producing a serving of food has half the environmental impact of vegetables, while a relative environmental impact of 2 indicates that producing a serving of food has twice the environmental impact of vegetables.

To examine the averaged relative environmental impact of food production (Fig. 3) across all indicators, we averaged the relative environmental impact of a food across all five environmental indicators examined here. For example, if a food has a relative environmental impact of 2 for GHGs, 3 for land, 10 for eutrophication, 6 for acidification, and 4 for scarcity weighted water use, the averaged relative environmental impact of that food would be 5 ([2 + 3 + 10 + 6 + 4] / 5).

Because we place equal weight on the 5 environmental indicators, we inherently assume that each environmental indicator is equally important. Other weightings, for instance weighting by proximity of current impacts to international environmental sustainability targets (e.g. the SDGs or the Paris Climate Agreement (10, 11)), could be useful to explore in further applications.

Spearman's Ranked Correlations

To examine the correlation between the pairwise combinations of the 5 health outcomes and 5 environmental indicators, we used Spearman's Ranked Correlations tests using the R function "rcorr" from the package "Hmisc". As used here, Spearman's Ranked Correlation tests examine whether the ranked impacts (1 = lowest) of consuming a food are correlated. As such, significant P-Values ($P < 0.05$) indicate that a food that has among the largest health benefit (or smallest environmental impact) for one outcome also has among the largest health benefit (or smallest environmental impact) for another outcome.

Of the 10 pairwise correlations between the 5 health outcomes, 8 Spearman's Ranked Correlation tests were significant at $P < 0.05$ (Table S2 in the SI Appendix). Of the 10 pairwise correlations between the 5 environmental outcomes, 9 Spearman's Ranked Correlation tests were significant at $P < 0.05$ (Table S2 in the SI Appendix).

Additional discussion of each food group

Whole grain cereals:

In dose-response meta-analyses, consuming an additional serving per day of whole grain cereals (30g dry weight) has been associated with a significant reduction in risk of total mortality, CHD, type II diabetes, and colorectal cancer, but not of stroke (23, 24, 27, 28). Consuming an additional serving of whole grain

cereals has among the largest health benefit for total mortality and type II diabetes of all foods included in this analysis (Fig. 1 and Fig. S1 in the SI Appendix). However, the health benefit of whole grain cereals is often non-linear, with the potential health benefits of a second (or third) serving being smaller than the first (or second serving). As such, large health benefits are often observed when increasing consumption of whole grain cereals when < 100g/day is consumed, while smaller health benefits are observed when increasing whole grain cereals when more than when > 100g/day is already consumed (28).

Per serving produced, whole grain cereals often have low mean environmental impacts, although there is large variation in the GHG emissions and water use per serving of whole grain cereals produced (Figs. 1–2, and Figs. S1–S2 in the SI Appendix). The variation in GHG emissions from whole grain cereals primarily results from differences between cereals. Rice production has 100–200% higher GHG emissions per serving produced than other cereals because methane, a greenhouse gas that has greater radiative forcing and thus warming potential than carbon dioxide, is released via anaerobic decomposition when rice paddies are flooded (15). There is also regional variation in the GHG emissions per serving of cereals produced, with higher GHG emissions resulting from production systems that use nutrients (fertilizer and manure) less efficiently or that are in regions with large carbon stores (e.g., peatlands) (40). Similarly, scarcity weighted water use per serving of whole grain cereal production, and for many other foods examined here, is highly variable, although is more dependent on the location of production rather than the type of cereal being produced. Scarcity weighted water use in whole grain cereal production, as well as production of other foods, is high in regions with limited water availability, such as North Africa, the Middle East, Central Asia, southeastern Australia, southwestern North America, and the west coast of South America, but is low in regions with large amounts of water availability, such as Amazonia, Southeast Asia, the eastern United States, and the United Kingdom (16).

Nuts:
"Nuts" includes both peanuts and tree nuts because consumption of peanuts and tree nuts has a similar impact on health outcomes. In meta-analyses, consuming an additional serving per day of nuts has been associated with significant reductions in total mortality and type II diabetes, but not for CHD, stroke, or colorectal cancer (23, 24, 27, 31) (Fig. 1). A significant reduction in risk of total mortality is observed even when small quantities of nuts are consumed. For instance, increasing nut consumption from 0 to 5g/day is associated with an approximately 7% reduction in risk of total mortality ($P < 0.05$) (24). As with whole grain cereals, the health benefit of nut consumption is non-linear, with smaller health benefits when > 30g/day of nuts are already consumed (24).

Nut production has low mean environmental impacts for GHG emissions, land use, acidification, and eutrophication (Figs. 1–2 and Figs. S1–2 in the SI Appendix). Scarcity weighted water use for nuts is highly variable because of variations in regional water availability where nuts are produced and because

producing different nuts uses different amounts of water. Chestnut and peanut production, for instance, uses small quantities of water (< 200 m^3 of irrigation water per tonne produced); hazelnut, walnut, and almond production uses intermediate amounts of water (from 1,000 to 2,000 m^3 of irrigation water per tonne produced); and pistachio production uses large quantities of water (> 7,000 m^3 of irrigation water per tonne produced) (59). Acidification resulting from nut production systems is also variable, largely because differences in fertilizer application rates and fertilizer use efficiencies in different cropping systems.

Legumes:

In dose-response meta-analyses, consuming an additional serving per day of legumes is not significantly associated with a change in any of the five health outcomes examined (23, 24, 27, 31) (Fig. 1). Analyses examining the extreme quantiles of legume consumption found that individuals who consumed the largest amount of legumes were at a significantly reduced risk of CHD and total mortality, but not stroke or colorectal cancer (23, 24, 27).

Producing a serving of legumes results in particularly low mean GHG emissions, acidification, and eutrophication (Figs. 1–2 and Figs. S1–2 in the SI Appendix). The GHG, acidification, and eutrophication impact of legume production is low because legumes have the ability to fix nitrogen (convert atmospheric nitrogen into nitrogen usable by the plant), which in turn reduces fertilizer input requirements, and resultantly the GHG emissions, acidification, and eutrophication impacts of legume production because these impacts often stem from fertiliser application and runoff.

Fruits:

In dose-response meta-analyses, consuming an additional serving per day of fruit has been associated with a significant reduction in risk of total mortality, CHD, stroke, and colorectal cancer, but not of type II diabetes (19, 22, 27) (Fig. 1). Individual fruits vary in their impact on health, with starchy fruits (e.g., bananas) being less beneficial to health than many other fruits (22). The health benefit of fruit consumption is non-linear, with smaller additional health benefits observed when > 300g/day of fruits are already consumed. However, additional health benefits from consuming fruits are often observed when consuming up to 800g/day (22).

Fruits have low mean environmental impacts for every environmental indicator examined, although there is large variation in scarcity weighted water use (Figs. 1–2 and Figs. S1–2 in the SI Appendix). Moreover, the method of fruit production is also a determinant of a fruit’s environmental impact. For instance, while fruit production primarily occurs in open fields, fruit production can also occur in heated greenhouses. Producing a serving of fruit in heated greenhouses emits 200% more GHG emissions but uses 25% the land of producing a serving of fruit in an open field (41). However, the GHG emissions of fruit produced in greenhouses could be reduced if energy is sourced from renewable energy sources.

Vegetables:

In dose-response meta-analyses, consuming an additional serving per day of vegetables has been associated with a significant reduction in risk of total mortality, CHD, stroke, and colorectal cancer, but not of type II diabetes (19, 22, 27) (Fig. 1). Individual vegetables may vary in their health benefit; leafy green vegetables such as spinach and kale are often associated with larger reductions in disease risk than many other types of vegetables, and have also been associated with a significant reduction in risk of type II diabetes (14, 22). The health benefit of vegetable consumption is non-linear, with smaller additional health benefits observed when > 300g/day of vegetables are already consumed. However, additional health benefits from consuming vegetables are often observed when consuming up to 800g/day of vegetables are consumed (22).

Vegetable production has low mean environmental impacts for each environmental indicator examined (Figs. 1–2 and Figs. S1–2 in the SI Appendix). There is, however, moderate variation around the mean impact for most environmental indicators, likely because of the diverse array of vegetables that are produced and consumed, but also because of regional differences in e.g. water availability or fertilizer application rates. However, as with fruits, vegetables can also be grown in heated greenhouses, which increases the GHG emissions but decrease the land use per serving of vegetables produced (41).

Potatoes:

Consuming a serving of potatoes (150g) per day has been significantly associated with increased risk of type II diabetes, but is not significantly associated with risk of total mortality, coronary heart disease, stroke, or colorectal cancer (26) (Fig. 1). Comparing the health outcomes of the highest and lowest potato consumers found that individuals who consumed the largest amount of potatoes was also not significantly associated with disease risk for coronary heart disease, stroke, colorectal cancer, or total mortality (26).

Potato production has low mean environmental impacts for most environmental indicators (Figs. 1–2 and Figs. S1–2 in the SI Appendix).

Refined grain cereals:

Consuming a serving per day (30g dry weight) of refined grain cereals has not been associated with a significant change in health risk for any of the health outcomes examined here (20, 23, 24, 27) (Fig. 1). Consuming larger quantities of refined grain cereals, however, may be detrimental for health. For instance, epidemiological studies that compared the health outcomes of individuals who consumed the largest quantity of refined grains with individuals that consumed the smallest quantity of refined grains found that individuals who consumed the largest quantity of refined grain cereals tended to be at increased risk of CHD (23). Similarly, consuming large quantities of white rice has been significantly associated with increased risk of type II diabetes (37).

Refined grain cereals have similar environmental impacts to whole grain cereals.

Eggs:

In dose-response meta-analyses, eggs have not been associated with a significant change in health for any of the five health outcomes examined here (21, 23, 24, 27) (Fig. 1). However, increasing egg consumption for individuals with pre-existing type II diabetes has been associated with a significant increase in risk of CHD mortality (51).

Egg production has low to intermediate mean environmental impacts for all five environmental indicators examined here (Figs. 1–2 and Figs. S1–2 in the SI Appendix). With the exception of water use, the variation in the environmental impact per serving of eggs produced is small.

Dairy:

In dose-response meta-analyses, dairy consumption has been associated with a significant decrease in risk of colorectal cancer, but not for total mortality or incidences of type II diabetes, stroke, or heart disease (18, 23, 24, 27) (Fig. 1). It is unclear whether skim and whole fat dairy products differ in their impact on health; the evidence that exists is limited and is often contradictory. See Mullie et al. (2016) (60) for a more in-depth discussion.

Producing a serving of dairy products has an intermediate environmental impact for GHG emissions, land use, acidification, and eutrophication, although there is considerable variation around the mean impact for each indicator (Figs. 1–2 and Figs. S1–2 in the SI Appendix). The 5th and 95th percentile scarcity weighted water use and eutrophication impacts of dairy production, for instance, vary by more than an order of magnitude.

Ruminants (e.g., cows, sheep, goats, and camels) are able to convert grasses and other low-protein, fibrous plant material into higher-protein and micronutrient rich human edible foods. This is particularly important in regions with limited access to markets and with limited arable land that is suitable for crop production. Furthermore, ruminant production is a major source of income for some of the more rural populations, particularly those in Eastern Africa (e.g., Kenya and Ethiopia). In regions where food production is inconsistent and where food insecurity is a constant threat, ruminant production for both meat and dairy can be an integral source of nutrition security (61).

Fish:

In dose-response meta-analyses, consuming an additional serving per day of fish has been associated with a significant reduction in risk of total mortality, CHD, and stroke, but not of type II diabetes or colorectal

cancer (17, 23, 24, 27) (Fig. 1). The health benefit of fish consumption is non-linear. For instance, smaller additional reductions in CHD mortality are observed by increasing fish consumption when > 50g/day of fish are already consumed (23).

The mean environmental impact per serving of fish produced varies across the environmental indicators examined (Figs. 1–2 and Figs. S1–2 in the SI Appendix). This is likely because the environmental impact of fish production differs by fish type and by production methodology (41). Wild-caught fish occupy no land, use minimal or no freshwater water, and results in very low acidification and eutrophication. However, wild-caught fish contribute to fishery depletion, with over 30% percent of fisheries currently being harvested unsustainably and 58% being fully fished (58). In contrast, producing a gram of aquaculture-raised fish occupies similar amounts of land, emits a similar amount of GHGs, and results in a similar amount of acidification as poultry production, but uses a similar amount of water and results in a similar amount of eutrophication as red meat. The GHG emissions of fish production are highly variable. Production of wild-caught fish via line, net seine, or midwater trawl fisheries, or aquaculture-raised fish by pond, net pen, or unfed aquaculture systems emits approximately one quarter the GHGs of bottom-trawling fisheries or recirculating aquaculture systems, respectively (41). Trawling fisheries also have higher rates of by-catch than other capture methodologies, while bottom-trawling fisheries also contribute to ecosystem degradation by dragging a net across the sea floor (62).

Chicken:

In dose-response meta-analyses, consuming an additional serving per day of chicken is not significantly associated with a reduction in total mortality or reductions in the incidences of type II diabetes, heart disease, or stroke (25, 30, 33) (Fig. 1). In some dose-response meta-analyses (33), but not in others (63), chicken consumption has been associated with significant reductions in colorectal cancer risk. However, the association between chicken consumption and colorectal cancer is complicated because of potential dietary confounding variables, such as the fact that consumption of red meat (which is associated with increased risk of colorectal cancer (27, 63)) often decreases when consumption of chicken increases. Ability (or lack thereof) to properly control for potential dietary confounding variables may influence the association between poultry consumption and colorectal cancer in specific (and other associations more generally), and partially explain why there is a lack of clarity around the association between chicken consumption and risk of colorectal cancer. While increasing consumption of chicken in the absence of other dietary changes is not associated with reduced mortality, substituting chicken for red or processed red meat has been associated with a significant reduction in risk of total mortality (64).

Producing a serving of chicken has higher mean environmental impacts than most other foods except fish and unprocessed and processed red meat (Figs. 1–2 and Figs. S1–2 in the SI Appendix). Producing a

serving of chicken has higher environmental impacts than most other foods because of the amount of feed required to produce it: on average, producing a gram of chicken requires 4.7 ± 0.27g of feed.

Unprocessed and processed red meat:

In dose-response meta-analyses, consumption of unprocessed and processed red meat (e.g., pig, beef, sheep, and goat meat) have both been associated with significant increases in disease risk for every health endpoint included in this analysis (23, 24, 27, 29) (Fig. 1). Despite the smaller average serving size of processed red meat (50g vs 100g for unprocessed red meat), processed red meat is often associated with larger increase in disease risk than is unprocessed red meat. This is likely because of the higher levels of nitrate, nitrate, and sodium in processed meats (65).

Unprocessed and processed red meat have the highest mean environmental impact of all foods examined here for most environmental indicators (Figs. 1–2 and Figs. S1–2 in the SI Appendix). The GHG emissions from red meat production are high largely because of the amount of feed required to produce red meat (5.7 ± 0.6g of feed per gram of edible pork; 14.5 ± 0.2g of feed per gram of edible sheep or goat meat; and 20.0 ± 0.8g of feed per gram of edible beef produced (66)), but also because ruminants produce methane when digesting food through a processed called enteric fermentation (66). In industrial (i.e., confined animal operation) livestock systems, red meat production has high environmental impacts for the other environmental indicators examined here largely because of the amount of feed required to produce red meat. While pasture-based ruminant production systems do not use as much or any concentrate feed as industrial operations, pasture-based ruminant meat does not necessarily have lower environmental impacts than ruminant meat from industrial systems (41). Lifetime methane emissions from pasture-raised ruminants are greater than those in industrial systems because pasture-raised ruminants live longer than ruminants produced in industrial systems. In addition, acidification and eutrophication from pasture-based ruminant systems can be particularly large, especially if the manure is not collected from the pastures and treated.

Sugar-sweetened beverages:

Dose-response meta-analyses have found that added sugars are significantly associated with an increased risk of CHD but not for total mortality (44, 45) (Fig 1). However, individual cohorts have found a positive association between added sugar consumption and risk of total mortality (44). While dose-response meta-analyses examining the association between added sugars and type II diabetes, stroke, and colorectal cancers do not yet exist, reviews have repeatedly shown that added sugar consumption is associated with increased risk of type II diabetes (e.g., ref (67)). Consuming a serving of Sugar-sweetened beverages each day is significantly associated with increased risk of type II diabetes, CHD, and stroke, but not with risk of total mortality or colorectal cancer risk of type II diabetes (e.g., ref (67)).

Producing a serving of added sugars or Sugar-sweetened beverages has among the lowest environmental impact for GHG emissions, acidification, and eutrophication (Figs. 1–2 and Figs. S2 in the SI Appendix), although scarcity weighted water use is highly variable and dependent on where the sugar is produced.

Olive oil:
Consuming an additional serving of olive oil per day has been associated with statistically significant reductions in risk of type II diabetes and stroke, but not for CHD (32, 68) (Fig. 1). While there is no data for the association between olive oil consumption and risk total mortality from dose-response meta-analyses, we estimated that the RR of total mortality of consuming an additional 10g serving of olive oil per day is 0.92 (range = 0.86 – 0.99) by using the RR of disease-specific endpoints (CHD, stroke, and diabetes) and their relative contributions to global mortality as estimated by the Global Burden of Disease (2) (see Methods in the SI Appendix). Consuming other oils high in polyunsaturated fatty acids and low in saturated fatty acids when consumed in place of hydrogenated oils has been associated with a significant reduction in risk of heart disease and total mortality (36, 69). Dose-response meta-analyses examining the association between olive oil consumption and colorectal cancer have not yet been published.

Producing a serving of olive oil production has low mean environmental impacts for each environmental indicator examined (Figs. 1–2 and Figs. S1–2 in the SI Appendix). As with many other foods, however, the scarcity weighted water use impact is highly variable and is dependent on total water availability where olives are produced.

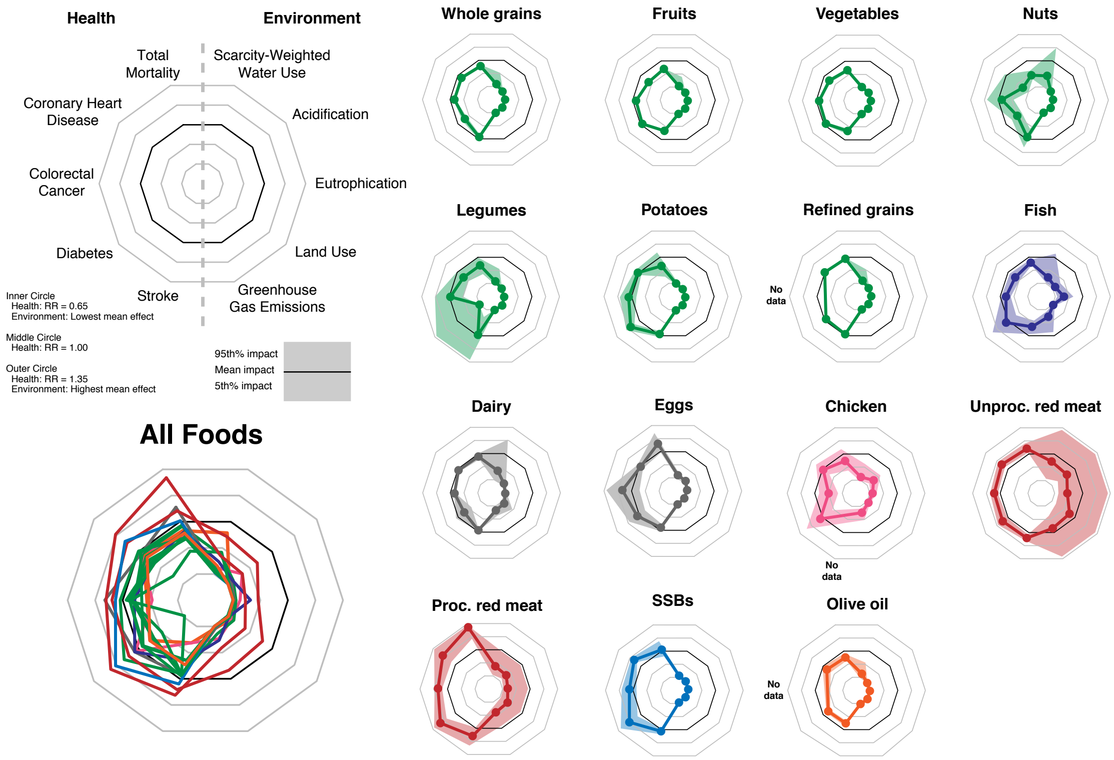

Fig. S1. Radar plots of relative health and environmental impacts per serving of food consumed per day. Solid line indicates mean impact per serving, and shading indicates 95% confidence intervals around the mean. Points closer to the origin are healthier, for the left-hand-side of each figure, and have lower environmental impacts, for the right-hand-side of each figure. For health outcomes, inner circle indicates a relative risk (RR) of 0.65 (or a 35% reduced risk of disease per additional serving consumed), middle circle indicates an RR of 1.00 (no change in disease risk), and outer circle indicates a RR of 1.35 (35% increased disease risk). For environmental outcomes, environmental impacts are plotted on a linear scale, where the inner circle indicates lowest mean impact per serving of food produced across the 15 foods examined, the outer circle indicates the highest mean impact, and the middle circle indicates environmental impacts that are halfway between the lowest and highest mean impact per serving (e.g., (lowest impact + highest impact) / 2). The "All Foods" radar plot contains data from the radar plots for the 15 food groups superimposed onto a single plot. Data used to create the plot is available in the Supplemental Data in the SI Appendix. Labels are ACM = total mortality; CHD = coronary heart disease; CRC = colorectal cancer; DIA = type II diabetes; STR = stroke; AP = acidification; EP = eutrophication; GHG = greenhouse gas emissions; LU = land use; and H2O = scarcity weighted water use. Indicators with strikethroughs indicate that data for the indicator and that food is not available (colorectal cancer and olive oil, colorectal cancer and refined grain cereals, and stroke and chicken). The association between total mortality and olive oil was estimated by weighting disease-specific contributions (e.g., CHD, stroke, and diabetes) to mortality by disease-specific relative risk (2).

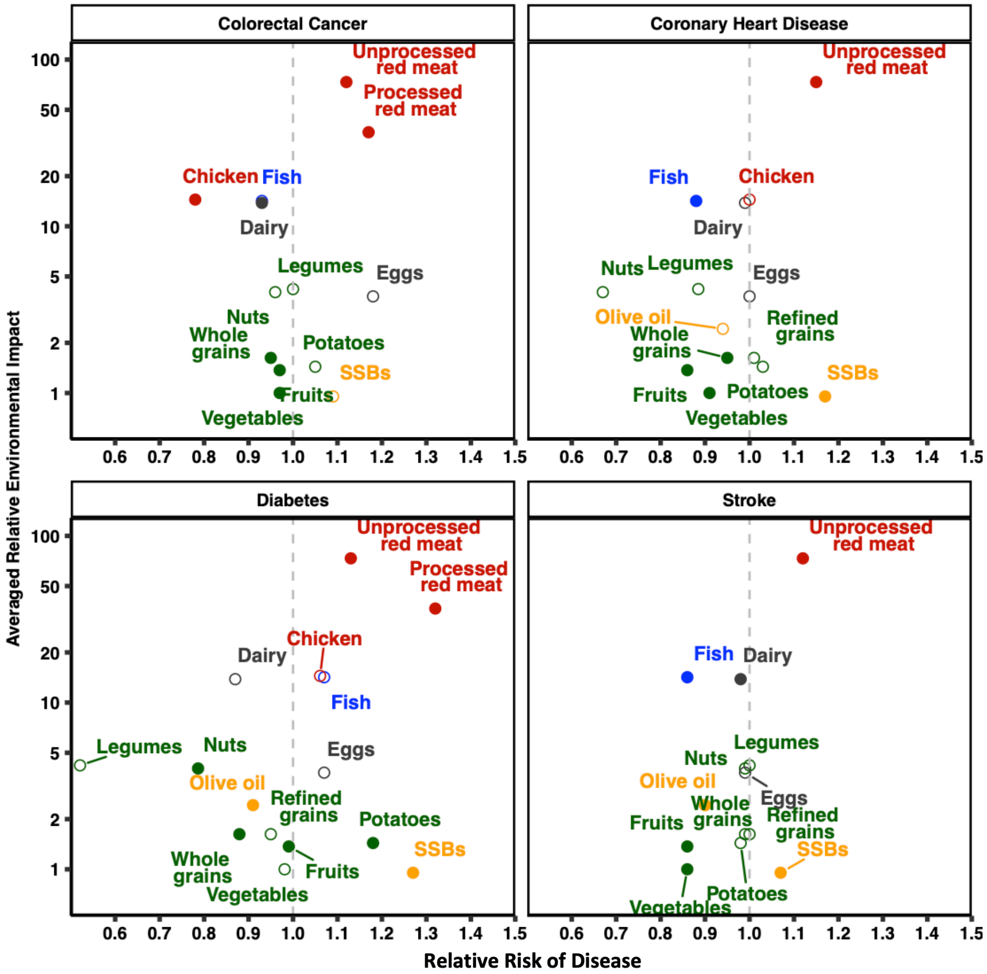

Fig. S2. Association between the health and the average relative environmental impacts of consuming an additional daily serving of each of 15 food groups. Each graph shows a different disease. Y-axis is on a log scale and is the AREI based on five environmental outcomes. X-axis is the relative risk of the listed disease, where a relative risk > 1 indicates that consuming an additional serving of a food group is associated with increased disease risk and a relative risk < 1 indicates that this consumption is associated with lowered disease risk. Labels and points are colored with green = minimally processed plant-based foods; dark blue = fish; black = dairy and eggs; pink = chicken; red = unprocessed red meat (beef, lamb, goat and pork) and processed red meat; dark yellow = sugar-sweetened beverages; and light yellow = olive oil. Food groups associated with a significant change in disease risk (at P < 0.05) are denoted by solid circles. Serving sizes: whole grains (30g dry weight); refined grains (30g dry weight); fruits (100g); vegetables (100g); nuts (28g); legumes (50g dry weight); potatoes (150g); fish (100g); dairy (200g); eggs (50g); chicken (100g); unprocessed red meat (100g); processed red meat (50g); Sugar-sweetened beverages (SSBs; 225g); olive

oil (10g). Data in SI Appendix.

Table S1

Lead Author	Year Published	Journal	Title
**Wallin**	2012	Diabetes Care	Fish consumption, dietary long-chain n-3 fatty acids, and risk of type 2 diabetes
**Aune**	2013	AJCN	Dairy products and the risk of type 2 diabetes: a systematic review and dose-response meta-analysis of cohort studies
**Aune**	2013	European Journal of Epidemiology	Whole grain and refined grain consumption and the risk of type 2 diabetes: A systematic review and dose-response meta-analysis of cohort studies
**Feskens**	2013	Current Diabetes Reports	Meat Consumption, Diabetes, and Its Complications
**Abete**	2014	Journal of Nutrition	Association between total, processed, red and white meat consumption and all-cause, CVD and IHD mortality: a meta-analysis of cohort studies
**Afshin**	2014	AJCN	Consumption of nuts and legumes and risk of incident ischemic heart disease , stroke , and diabetes : a systematic review and meta-analysis
**Martinez-Gonzalez**	2014	British Journal of Nutrition	Olive oil consumption and risk of CHD and/or stroke: a meta-analysis of case-control, cohort, and intervention studies
Shi	2014	European Journal of Nutrition	Dose-response meta-analysis of poultry intake and colorectal cancer incidence and mortality
**Imamura**	2015	BMJ	Consumption of sugar-sweetened beverages, artificially sweetened beverages, and fruit juice and incidence of type 2 diabetes: systematic review, meta-analysis, and estimation of population attributable fraction
**Wu**	2015	Nutrition, Metabolism & Cardiovascular Diseases	Fruit and vegetable consumption and risk of type 2 diabetes mellitus: A dose-response meta-analysis of propsective cohort studies
**Aune**	2016	BMJ	Whole grain consumption and risk of cardiovascular disease , cancer , and all cause and cause specific mortality : systematic review and dose-response meta-analysis of prospective studies
Wallin	2016	Diabetologia	Egg consumption and risk of type 2 diabetes: a prospective study and dose_response meta-analysis
**Aune**	2017	International Journal of Epidemiology	Fruit and vegetable intake and the risk of cardiovascular disease, total cancer and all-cause mortality_a systematic review and

			dose- response
Bechthold	2017	Critical Reviews in Food Science and Nutrition	Food groups and risk of coronary heart disease, stroke and heart failure: A systematic review and dose-response meta-analysis of prospective studies
**Mohammadi**	2017	Clinical Nutrition ESPEN	Dietary poultry intake and the risk of stroke: a dose-response meta-analysis of prospective cohort studies
Schwingshakl	2017	AJCN	Food groups and risk of all-cause mortality: a systematic review and meta-analysis of prospective studies
Schwingshakl	2017	Nutrition and Diabetes	Olive oil in the prevention and management of type 2 diabetes mellitus: a systematic review and meta-analysis of cohort studies and intervention trials
Schwingshakl	2018	International Journal of Cancer	Food groups and risk of colorectal cancer
Schwingshakl	2018	European Journal of Nutrition	Potatoes and risk of chronic disease: a systematic review and dose-response meta-analysis

Table S1. Dose-response health analyses used in this analysis.

Table S2

Health			Environment		
Health Outcome 1	Health Outcome 2	P-Value	Environmental Impact 1	Environmental Impact 2	P-Value
Total Mortality	Coronary Heart Disease	0.005	Acidification Potential	Eutrophication Potential	<.001
Total Mortality	Colorectal Cancer	0.139	Acidification Potential	GHG Emissions	0.002
Total Mortality	Type II Diabetes	0.027	Acidification Potential	Land Use	<.001
Total Mortality	Stroke	0.035	Acidification Potential	Scarcity Weighted Water Use	0.008
Coronary Heart Disease	Colorectal Cancer	0.021	Eutrophication Potential	GHG Emissions	<.001
Coronary Heart Disease	Type II Diabetes	0.002	Eutrophication Potential	Land Use	<.001
Coronary Heart Disease	Stroke	0.008	Eutrophication Potential	Scarcity Weighted Water Use	0.015
Colorectal Cancer	Type II Diabetes	0.022	GHG Emissions	Land Use	0.034
Colorectal Cancer	Stroke	0.027	GHG Emissions	Scarcity Weighted Water Use	0.145
Type II Diabetes	Stroke	0.294	Land Use	Scarcity Weighted Water Use	<.001

Table S2. P-values of the Spearman's Ranked Correlations between the pairwise health and environmental outcomes. All correlations are positive.

Table S3

Food Group	Mortality	CHD (Coronary Heart Disease)	Colorectal Cancer	Stroke	Diabetes
**Chicken**	100	100	100	NA	100
**Dairy**	200	200	200	200	200
**Eggs**	50	50	50	50	21.4
**Fish**	100	100	100	100	100
**Fruits**	100	100	100	100	100
**Legumes**	50	50	50	50	50
**Nuts**	28	28	28	28	28
**Olive oil**	10	10	NA	10	10
**Potatoes**	150	150	150	150	150
**Processed meat**	50	50	50	50	50
**Red meat**	100	100	100	100	100
**Refined grains**	30	30	NA	30	30
**Sugar-sweetened beverages**	25	25	25	25	25
**Vegetables**	100	100	100	100	100
**Whole grains**	30	30	30	30	30

Table S3. Serving sizes, as grams per serving, as reported in the dose-response health meta-analyses (Table S1), and thus as used in this analysis. Serving sizes for refined grain cereals and whole grain cereals are reported in dry weight; serving size for Sugar-sweetened beverages (sugar-sweetened beverages) is reported as grams of sugar. "NA" indicates that health data for the food group and health outcome were not available from dose response meta-analyses.

Table S4

Food Group	Disease	Asia	Canada/USA	Europe	Oceania	Other or Not Specified
Chicken	ACM	23% (30%)	32% (39%)	45% (32%)	-	-
	CHD	27% (32%)	31% (38%)	42% (30%)	-	-
	CRC	4% (3%)	47% (45%)	43% (47%)	6% (5%)	-
	Stroke	40% (61%)	60% (39%)	-	-	-
Dairy	ACM	33% (33%)	14% (14%)	49% (49%)	0% (0%)	4% (4%)
	CHD	0% (0%)	50% (49%)	50% (51%)	-	-
	CRC	6% (7%)	48% (47%)	46% (46%)	-	-
	Diabetes	7% (14%)	84% (76%)	8% (9%)	1% (2%)	-
	Stroke	-	78% (72%)	22% (28%)	-	-
Eggs	ACM	6% (6%)	10% (10%)	84% (84%)	-	-
	CHD	0% (0%)	50% (49%)	50% (51%)	-	-
	CRC	53% (78%)	-	47% (22%)	-	-
	Diabetes	10% (24%)	22% (20%)	68% (56%)	-	-
	Stroke	-	78% (72%)	22% (28%)	-	-
Fish	ACM	33% (33%)	16% (16%)	50% (50%)	1% (1%)	-
	CHD	0% (0%)	50% (49%)	50% (51%)	-	-
	CRC	12% (13%)	43% (33%)	44% (52%)	1% (2%)	-
	Diabetes	44% (44%)	50% (50%)	6% (6%)	-	-
	Stroke	-	78% (72%)	22% (28%)	-	-
Fruits	ACM	14% (18%)	9% (6%)	73% (67%)	5% (9%)	-
	CHD	32% (36%)	30% (28%)	37% (35%)	0% (1%)	-
	CRC	8% (8%)	56% (64%)	37% (28%)	-	-
	Diabetes	9% (24%)	88% (73%)	3% (4%)	-	-
	Stroke	33% (38%)	40% (39%)	26% (23%)	-	-
Legumes	ACM	41% (41%)	0% (0%)	53% (53%)	0% (0%)	6% (6%)
	CHD	0% (0%)	50% (49%)	50% (51%)	-	-
	CRC	18% (19%)	34% (46%)	48% (35%)	-	-

	Diabetes	58% (64%)	42% (36%)	-	-	-
	Stroke	-	78% (72%)	22% (28%)	-	-
Nuts	ACM	21% (21%)	27% (27%)	48% (48%)	0% (0%)	4% (4%)
	CHD	0% (0%)	50% (49%)	50% (51%)	-	-
	CRC	3% (2%)	71% (58%)	26% (40%)	-	-
	Diabetes	8% (20%)	88% (77%)	3% (2%)	-	-
	Stroke	-	78% (72%)	22% (28%)	-	-
Olive Oil	CHD	-	-	100% (100%)	-	-
	Diabetes	-	87% (78%)	13% (22%)	-	-
	Stroke	-	-	100% (100%)	-	-
Potatoes	ACM	-	50% (39%)	50% (61%)	-	-
	CHD	-	40% (42%)	60% (58%)	-	-
	CRC	6% (5%)	41% (57%)	53% (38%)	-	-
	Diabetes	6% (18%)	87% (66%)	5% (8%)	3% (9%)	-
	Stroke	-	50% (53%)	50% (47%)	-	-
Processed Red Meat	ACM	-	53% (58%)	47% (42%)	-	-
	CHD	4% (3%)	50% (55%)	46% (42%)	-	-
	CRC	6% (7%)	60% (57%)	33% (35%)	2% (2%)	-
	Diabetes	-	-	-	-	100% (100%)
	Stroke	-	78% (72%)	22% (28%)	-	-
Red Meat	ACM	20% (26%)	42% (42%)	38% (32%)	-	-
	CHD	18% (22%)	43% (44%)	40% (34%)	-	-
	CRC	11% (13%)	63% (58%)	24% (28%)	2% (1%)	-
	Diabetes	-	-	-	-	100% (100%)
	Stroke	-	78% (72%)	22% (28%)	-	-
Refined Grains	ACM	2% (2%)	98% (98%)	-	-	-
	CHD	0% (0%)	50% (49%)	50% (51%)	-	-
	Diabetes	-	80% (84%)	20% (16%)	-	-
	Stroke	-	78% (72%)	22% (28%)	-	-
Sugar-sweetened beverages	ACM	7% (7%)	58% (58%)	35% (35%)	-	-
	CHD	0% (0%)	50% (49%)	50% (51%)	-	-
	CRC	-	25% (15%)	-	-	75% (85%)
	Diabetes	9% (17%)	75% (65%)	16% (18%)	-	-
	Stroke	-	78% (72%)	22% (28%)	-	-
Vegetables	ACM	15% (19%)	9% (6%)	71% (65%)	5% (10%)	-
	CHD	11% (9%)	56% (61%)	33% (30%)	0% (1%)	-
	CRC	9% (9%)	54% (63%)	37% (28%)	-	-
	Diabetes	19% (40%)	73% (54%)	7% (6%)	-	-
	Stroke	19% (17%)	34% (39%)	47% (44%)	-	-

Whole Grains	ACM	0% (0%)	70% (70%)	30% (30%)	-	-
	CHD	0% (0%)	50% (49%)	50% (51%)	-	-
	CRC	-	68% (73%)	32% (27%)	-	-
	Diabetes	-	91% (90%)	9% (10%)	-	-
	Stroke	-	78% (72%)	22% (28%)	-	-

Table S4. Geographic distribution of study participants in the dose-response meta-analyses used here. Estimates are divided by food group and disease outcome, and are reported for person years (number of participants).

Additional data table S1 (separate file)

Additional Data Table S1 contains all of the data used to make Figs. 1–4 and Fig. S1.

References

1. Forouzanfar M, et al. (2015) Global , regional , and national comparative risk assessment of 79 behavioural , environmental and occupational , and metabolic risks or clusters of risks in 188 countries , 1990 – 2013 : a systematic analysis for the Global Burden of Disease Study 2013. *Lancet* 386:2287–1323.
2. IHME (2018) GBD Compare. Available at: https://vizhub.healthdata.org/gbd-compare/ [Accessed May 14, 2018].
3. Foley J a, et al. (2011) Solutions for a cultivated planet. *Nature* 478(7369):337–42.
4. IPCC (2014) *Climate Change 2014: Synthesis Report. Contribution of Working Groups I, II and III to the Fifth Assessment Report of the Intergovernmental Panel on Climage Change* (Geneva, Switzerland).
5. FAO (2019) No Title. Available at: www.faostat.fao.org.
6. Vitousek PM, et al. (1997) Human Alteration of the Global Nitrogen Cycle : Sources and Consequences Published by : Ecological Society of America Stable URL : http://www.jstor.org/stable/2269431. *Ecol Appl* 7(November 1996):737–750.
7. Molden D (2007) Comprehensive Assessment of Water Management in Agriculture. *Water for Food, Water for Life: A Comprehensive Assessment of Water Management in Agriculture* (Earthscan, International Water Management Institute, London, Colombo).
8. Ceballos G, et al. (2015) Accelerated modern human – induced species losses: entering the sixth mass extinction. *Sci Adv* 1(e1400253):1–5.
9. Lelieveld J, Evans JS, Fnais M, Giannadaki D, Pozzer A (2015) The contribution of outdoor air pollution sources to premature mortality on a global scale. *Nature* 525(7569):367–371.
10. Assembly UNG (2015) *Transforming Our World: The 2030 Agenda for Sustainable Development*.
11. United Nations Treaty Collection (2015) *Paris Agreement*.
12. Tilman D, Clark M (2014) Global diets link environmental sustainability and human health. *Nature* 515(7528):518–522.
13. Springmann M, Godfray HCJ, Rayner M, Scarborough P (2016) Analysis and valuation of the health and climate change cobenefits of dietary change. *Proc Natl Acad Sci U S A* 113(15):4146–4151.
14. Li M, Fan Y, Zhang X, Hou W, Tang Z (2014) Fruit and vegetable intake and risk of type 2 diabetes mellitus: Meta-analysis of prospective cohort studies. *BMJ Open* 4. doi:10.1136/bmjopen-2014-005497.
15. Poore J, Nemecek T (2018) Reducing food ' s environmental impacts through producers and

consumers. *Science (80-)* 992(June):987–992.
16. Boulay AM, et al. (2018) The WULCA consensus characterization model for water scarcity footprints: assessing impacts of water consumption based on available water remaining (AWARE). *Int J Life Cycle Assess* 23(2):368–378.
17. Wallin A, et al. (2012) Fish Consumption, Dietary Long-Chain n-3 Fatty Acids, and Risk of Type 2 Diabetes. *Diabetes Care* 35:918–29.
18. Aune D, Norat T, Romundstad P, Vatten LJ (2013) Dairy products and the risk of type 2 diabetes : a systematic review and dose-response meta-analysis of cohort studies 1 – 3. *Am J Clin Nutr* (6). doi:10.3945/ajcn.113.059030.
19. Wu Y, Zhang D, Jiang X, Jiang W (2015) Fruit and vegetable consumption and risk of type 2 diabetes mellitus : A dose-response meta-analysis of prospective cohort studies. *Nutr Metab Cardiovasc Dis* 25:140–147.
20. Aune D, et al. (2016) Whole grain consumption and risk of cardiovascular disease , cancer , and all cause and cause specific mortality : systematic review and dose-response meta-analysis of prospective studies. *Br Med J* 353:1–14.
21. Wallin A, Forouhi NG, Wolk A, Larsson SC (2016) Egg consumption and risk of type 2 diabetes : a prospective study and dose – response meta-analysis. *Diabetologia* 59:1204–1213.
22. Aune D, et al. (2017) Fruit and vegetable intake and the risk of cardiovascular disease, total cancer and all-cause mortality-a systematic review and dose-response meta-analysis of propsective studies. *Int J Epidemiol*:1–28.
23. Bechthold A, et al. (2017) Food groups and risk of coronary heart disease, stroke and heart failure: A systematic review and dose-response meta-analysis of prospective studies. *Crit Rev Food Sci Nutr* 0(0):1–20.
24. Schwingshackl L, et al. (2017) Food groups and risk of all-cause mortality: a systematic review and meta-analysis of prospective studies. *AJCN* 105:1462–73.
25. Mohammadi H, Jayedi A, Ghaedi E, Golbidi D, Shab-bidar S (2018) Dietary poultry intake and the risk of stroke: A dose–response meta-analysis of prospective cohort studies. *Clin Nutr ESPEN* 23:25–33.
26. Schwingshackl L, Schwedhelm C, Hoffmann G, Boeing H (2018) Potatoes and risk of chronic disease: a systematic review and dose–response meta-analysis. *Eur J Nutr* 0(0):1–9.
27. Schwingshackl L, et al. (2018) Food groups and risk of colorectal cancer. *Int J Cancer* 142(9):1748–1758.
28. Aune D, Norat T, Romundstad P, Vatten LJ (2013) Whole grain and refined grain consumption and the risk of type 2 diabetes: A systematic review and dose-response meta-analysis of cohort studies. *Eur J Epidemiol* 28(11):845–858.
29. Feskens EJM, Sluik D, van Woudenbergh GJ (2013) Meat Consumption , Diabetes , and Its Complications. *Curr Diab Rep* 13:298–306.
30. Abete I, Romaguera D, Vieira AR, Lopez de Munain A, Norat T (2014) Association between total, processed, red and white meat consumption and all-cause, CVD and IHD mortality: a meta-analysis of cohort studies. *Br J Nutr* 112(05):762–775.
31. Afshin A, Micha R, Khatibzadeh S, Mozaffarian D (2014) Consumption of nuts and legumes and risk of incident ischemic heart disease , stroke , and diabetes : a systematic review and meta-analysis. *Am J Clin Nutr* 100:278–289.
32. Martinez-Gonzalez MA, Dominguez LJ, Delgado-Rodriguez M (2014) Olive oil consumption and risk of CHD and / or stroke : a meta-analysis of case – control , cohort and intervention studies. *Br J Nutr* 112:248–259.
33. Shi Y, Yu PW, Zeng DZ (2014) Dose–response meta-analysis of poultry intake and colorectal cancer incidence and mortality. *Eur J Nutr* 54(2Dose–response meta–analysis of poultry intake and colorectal cancer incidence and mortality):243–250.
34. Imamura F, et al. (2015) Consumption of sugar-sweetened beverages, artificially sweetened beverages, and fruit juice and incidence of type 2 diabetes: systematic review, meta-analysis, and estimation of population attributable fraction. *Bmj*:h3576.
35. de Souza RJ, et al. (2015) Intake of saturated and trans unsaturated fatty acids and risk of all cause mortality, cardiovascular disease, and type 2 diabetes: systematic review and meta-analysis of observational studies. *Bmj*:h3978.
36. Jakobsen MU, et al. (2009) Major types of dietary fat and risk of coronary heart disease : a pooled

analysis of 11 cohort studies 1 – 3. *Am J Clin Nutr* 89:1425–1433.
37. Sun Q, et al. (2010) White rice, brown rice, and risk of type 2 diabetes in US Men and women. *Arch Intern Med* 170(11):961–969.
38. Liu S, et al. (2000) A Prospective Study of Whole-Grain Intake and Risk of Type 2 Diabetes Mellitus in US Women. *Am J Public Health* 90(9):1409–1415.
39. Herrero M, et al. (2013) Biomass use, production, feed efficiencies, and greenhouse gas emissions from global livestock systems. *Proc Natl Acad Sci U S A* 110(52):20888–93.
40. Carlson KM, et al. (2016) Greenhouse gas emissions intensity of global croplands. *Nat Clim Chang* 7(21 November). doi:10.1038/nclimate3158.
41. Clark M, Tilman D (2017) Comparative analysis of environmental impacts of agricultural production systems, agricultural input efficiency, and food choice. *Environ Res Lett* 12.
42. Pan A, et al. (2012) Red Meat Consumption and Mortality: Results From 2 Prospective Cohort Studies. *Arch Intern Med* 172(7):555–563.
43. Schnabel L, et al. (2018) Functional GI Disorders Association Between Ultra-Processed Food Consumption and Functional Gastrointestinal Disorders : Results From the French NutriNet-Santé Cohort. *Am J Gastroenterol* 113:1217–1228.
44. Yang Q, et al. (2014) Added Sugar Intake and Cardiovascular Diseases Mortality Among US Adults. *JAMA Intern Med* 174(4):516.
45. Tasevska N, et al. (2014) Sugars and risk of mortality in the NIH-AARP Diet and Health Study 1 – 4. *Am J Clin Nutr* 99:1077–88.
46. Müller O, Krawinkel M (2005) Malnutrition and health in developing countries. *CMAJ* 173(3):279–86.
47. IUCN (2017) No Title. *IUCN Red List Threat Species Version 2016-5*.
48. Drewnowski A, Popkin BM (1997) The nutrition transition : New trends in the global diet. *Nutr Rev* 55(2):31–43.
49. Springmann M, et al. (2018) Options for keeping the food system within environmental limits. *Nature*. doi:10.1038/s41586-018-0594-0.
50. Willett W, et al. (2019) Food in the Anthropocene : the EAT – Lancet Commission on healthy diets from sustainable food systems. *Lancet* 6736(18). doi:10.1016/S0140-6736(18)31788-4.
51. Rong Y, et al. (2013) Egg consumption and risk of coronary heart disease and stroke : dose-response meta-analysis of. *BMJ* 346(8539):1–13.
52. Khan NA, et al. (2011) Ethnicity and Sex Affect Diabetes Incidence and Outcomes. *Diabetes Care* 34:96–101.
53. Siegel RL, Miller KD, Jemal A (2017) Cancer Statistics, 2017. *CA Cancer J Clin* 67(1):7–30.
54. Zheng J, et al. (2012) Fish consumption and CHD mortality: an updated meta-analysis of seventeen cohort studies. *Public Health Nutr* 15(04):725–737.
55. Aune D, et al. (2016) Nut consumption and risk of cardiovascular disease, total cancer, all-cause and cause-specific mortality: a systematic review and dose-response meta-analysis of prospective studies. *BMC Med* 14(1):207.
56. Whitlock G, et al. (2009) Body-mass index and cause-specifi c mortality in 900 000 adults : collaborative analyses of 57 prospective. *Lancet* 373(9669):1083–1096.
57. Seufert V, Ramankutty N (2017) Many shades of gray — The context-dependent performance of organic agriculture. *Sci Adv* 3(March):1–14.
58. FAO (2016) *The State of World Fisheries and Aquaculture: Contributing to food security and nutrition for all*.
59. Mekonnen MM, Hoekstra AY (2011) The green, blue and grey water footprint of crops and derived crop products. *Hydrol Earth Syst Sci* 15(5):1577–1600.
60. Mullie P, Pizot C, Autier P (2016) Daily milk consumption and all-cause mortality, coronary heart disease and stroke: a systematic review and meta- analysis of observational cohort studies. *BMC Public Health* 16(1236):1–8.
61. Smith J, et al. (2013) Beyond milk, meat, and eggs: Role of livestock in food and nutrition security. *Anim Front* 3(1):6–13.
62. Dayton PK, Thrush SF, Agardy MT, Hofman RJ (1995) Environmental effects of marine fishing. *Aquat Conserv Mar Freshw Ecosyst* 5(March):205–232.
63. Vieira AR, et al. (2017) Foods and beverages and colorectal cancer risk : a systematic review and meta-analysis of cohort studies , an update of the evidence of the WCRF-AICR Continuous Update

Project. *Ann Oncol* 28:1788–1802.

64. Pan A, et al. (2012) Red meat consumption and mortality: results from 2 prospective cohort studies. *Arch Intern Med* 172(7):555–63.
65. Etemadi A, et al. (2017) Mortality from different causes associated with meat, heme iron, nitrates, and nitrites in the NIH-AARP Diet and Health Study: population based cohort study. *Bmj*:j1957.
66. Tilman D, Clark M (2014) Global diets link environmental sustainability and human health. *Nature* 515:518–22.
67. Hu FB (2011) Globalization of diabetes: The role of diet, lifestyle, and genes. *Diabetes Care* 34(6):1249–1257.
68. Schwingshackl L, et al. (2017) Olive oil in the prevention and management of type 2 diabetes mellitus : a systematic review and meta-analysis of cohort studies and intervention trials. *Nutr Diabetes* 7(3):e262-6.
69. Wang DD, et al. (2016) Association of specific dietary fats with total and cause-specific mortality. *JAMA Intern Med* 176(8):1134–1145.
